# Cholesterol Metabolism: An Ally in the Development and Progression of Cervical Cancer

**DOI:** 10.3390/ijms27020591

**Published:** 2026-01-06

**Authors:** Imelda Martínez-Ramírez, J. Omar Muñoz-Bello, Adriana Contreras-Paredes, Elías Parra-Hernández, Adela Carrillo-García, Marcela Lizano

**Affiliations:** 1 Subdirección de Investigación Básica, Unidad de Investigación Biomédica en Cáncer, Instituto Nacional de Cancerología, Mexico City 14080, Mexico; imartinezr@incan.edu.mx (I.M.-R.); jmunozb@incan.edu.mx (J.O.M.-B.); acontrerasp@incan.edu.mx (A.C.-P.); adcarrillo2004@yahoo.com.mx (A.C.-G.); 2 Gerencia de Análisis y Desarrollo de Pruebas Biológicas, Comisión de Control Analítico y Ampliación de Cobertura (CCAYAC)-Comisión Federal para la Protección Contra Riesgos Sanitarios (COFEPRIS), Mexico City 14050, Mexico; eparra@cofepris.gob.mx; 3 Departamento de Medicina Genómica y Toxicología Ambiental, Instituto de Investigaciones Biomédicas, Universidad Nacional Autónoma de México, Ciudad Universitaria, Mexico City 04510, Mexico

**Keywords:** cervical cancer, Human Papillomavirus, cholesterol metabolism, HPV oncoproteins, therapeutic approaches, biomarkers

## Abstract

Despite screening programs and vaccination campaigns, cervical cancer (CC) remains a health problem worldwide. The involvement of the E6 and E7 oncoproteins of Human Papillomavirus (HPV) is crucial for the development and progression of this type of cancer. Metabolic reprogramming by cancer cells has gained relevance in the last decade due to its ability to promote cell growth, survival, invasion, metastasis, and resistance to therapy. In this review, we focus on alterations in cholesterol metabolism that significantly influence the development and progression of CC, as well as the clinical outcome of patients. Furthermore, evidence from comprehensive omics studies suggesting that E6 and E7 are involved in the exacerbation of elements related to cholesterol metabolism is analyzed. Preclinical and clinical studies are also discussed that demonstrate that cholesterol metabolism is a potential therapeutic target, highlighting its impact on reducing tumor growth, altering the tumor microenvironment, and improving antitumor immunity.

## 1. Introduction

Cervical cancer (CC) continues to be a health problem in the female population, particularly in developing countries. Although the introduction of Human Papillomavirus (HPV) vaccination has shown effectiveness in reducing CC cases in some countries [1,2], this type of cancer continues to rank fourth in cancer mortality in the female population worldwide [3], which is partly explained by the fact that it is commonly diagnosed at advanced stages [4]. Therefore, there is a need to improve therapeutic strategies, which requires a deeper understanding of the biological processes involved in CC.

The main etiologic agent for the development of cervical cancer is the persistent infection with high-risk HPV types, which are responsible for almost all cases of cervical cancer [5]. It has been shown that for cancer phenotype maintenance, the continued expression of the HPV E6 and E7 oncogenes is required [6]. HPV oncoproteins contribute to CC development through their interactions with several cellular proteins, affecting essential processes such as proliferation, DNA damage repair, the immune system, apoptosis, and metabolic pathways [7,8,9].

Cancer cells reprogram their cellular metabolism to meet bioenergetic and biosynthetic needs and can also alter the metabolism of the tumor microenvironment [10]. Increased cancer cell proliferation rates lead to a clear shortage and increased demand for nutrients such as glucose, glutamine, and lipids, resulting in increased use of these carbon sources for anabolic processes. Reprogramming of cell metabolism provides cancer cells growth advantages, which result in increased cell survival, invasion, metastasis, and resistance to chemo and radiotherapy [11]. It has been shown that HPV E6 and E7 oncoproteins regulate key enzymes of the glycolytic pathway, promoting the Warburg effect [12,13,14]. Some insights have also been gained into the regulation of glutaminolysis by HPV oncoproteins [8]. Nevertheless, their participation in other metabolic switches has not been studied in depth.

Lipid metabolism provides cells with energy, membrane production, and activates lipids-mediated signaling pathways [15,16]. Among the different types of lipids, cholesterol is a key component for maintaining cellular homeostasis [17]. Cholesterol is essential for cell growth and development, being an important component of the mammalian cell membrane that ensures membrane fluidity to maintain the structural integrity of the cell [17]. It also participates in the production of steroid hormones, vitamins, bile acids, and the generation of eicosanoids [18]. Therefore, maintaining its homeostasis must be tightly regulated, since an imbalance in any part of its metabolism has been associated with pathological conditions such as cancer [19,20].

Alterations in factors implicated in cholesterol synthesis promote the development and progression of different types of cancers, significantly affecting apoptosis, proliferation, migration, and invasion [21,22,23,24,25]. This article analyzes how changes in cholesterol metabolism in CC contribute to cancer progression and the clinical outcome of patients. Furthermore, the involvement of HPV oncoproteins E6 and E7 in the dysregulation of genes and proteins involved in cholesterol metabolism is explored. The article also reviews therapeutic strategies targeting exacerbated cholesterol metabolism and how combining these strategies with conventional therapy can exert promising anticancer effects, focused on improving the quality of life of patients with CC.

## 2. Homeostasis of Cholesterol Metabolism

Cholesterol is one of the three most important cellular membrane lipids, making up almost 25% of it. Furthermore, it is an essential structural molecule for maintaining cellular homeostasis [17,20]. Cholesterol is distributed across cellular membranes and interacts with other lipids and proteins, maintaining membrane permeability and fluidity. This ensures structural integrity and cell functions including cell adhesion, signal transduction, membrane trafficking, and interactions with pathogens [20,26,27]. Besides its role as a cellular membrane component, cholesterol acts as a precursor of steroid hormones, vitamins, oxysterols, and bile acids and participates in intracellular signal pathways, contributing to important physiological processes [18]. At the intracellular level, the homeostasis of cholesterol is tightly monitored and regulated by a complex network that controls cholesterol biosynthesis, metabolism, and intracellular transport [28].

Most mammalian cells obtain cholesterol via endogenous synthesis or by exogenous uptake. The endogenous synthesis converts acetyl-CoA into cholesterol in the cytoplasm and endoplasmic reticulum by over 20 enzymatic reactions, that can be grouped into the following steps: condensation of acetyl-CoA to 3-hydroxy-3-methylglutaryl-CoA (HMG-CoA); reduction of HMG-CoA to mevalonate; conversion of mevalonate into isopentenyl pyrophosphate and farnesyl pyrophosphate; condensation of farnesyl pyrophosphate into squalene; oxygenation and cyclization of squalene to lanosterol, which is finally converted to cholesterol. After synthesis, the newly formed cholesterol is transported to the cell membrane through the Golgi apparatus [23,29].

In the exogenous uptake pathway, cholesterol is taken up by cells through endocytosis via receptor-mediated mechanisms. Most cholesterol is stored as cholesterol esters (CE) and is transported in the body bound to different Apo proteins, forming a lipoprotein complex. Among these, low-density lipoprotein (LDL) is the main cholesterol transporter in the form of LDL-cholesteryl ester (LDL-CE), while high-density lipoprotein (HDL) transports cholesterol from peripheral tissues to the liver in the form of HDL-cholesteryl ester (HDL-CE) [30,31]. LDL receptor (LDLR)-mediated endocytosis is the most important mechanism whereby cells uptake cholesterol from LDL. The LDLR-LDL-CE complex is internalized into the cell via clathrin-coated pits, fusing with early endosomes where LDL-CE is dissociated from LDLR, and is then transferred to lysosomes, where the CE in the LDL is hydrolyzed through acid lipase hydrolysis to release the free cholesterol [31]. Free cholesterol is transported out of the lysosome by the Niemann-Pick type C proteins (NPC2 in the lysosome lumen, NPC1 in the lysosome membrane) [32,33]. Finally, cholesterol is exported to other membranes and incorporated into the metabolically active intracellular pool, with the help of sterol transfer proteins [17]. HDL uptake is a more complex and versatile mechanism that involves scavenger receptor B-type I (SR-BI). SR-B1 forms a water-free channel on the cell surface through which HDL-CE molecules diffuse to the cell plasma membrane. The CE molecule binds to HDL through its extracellular domain and selectively delivers it into the cell. The extracellular domain contains highly conserved cysteine residues essential for SR-B1 activity [34,35,36]. SR-B1 mediates bidirectional cholesterol transport between HDL and cells, delivering cholesteryl esters into cells from HDL and transporting free cholesterol out of cells into HDL. Also, SR-B1 redistributes cholesterol within the plasma membrane by increasing its availability to the extracellular space [37].

The regulation of cholesterol homeostasis occurs through a complex interplay at the transcriptional and post-transcriptional levels, enabling rapid adaptation to cellular needs. This process involves the sterol regulatory element binding protein 2 (SREBP-2), the nuclear erythroid 2-related factor 1 (NRF1), and the liver X receptor (LXR), which control cholesterol levels in the endoplasmic reticulum [38,39,40]. The accumulation of cholesterol and oxysterols inactivates the SREBP-2 pathway by promoting insulin-induced gene (INSIG)-mediated retention of the SCAP (SREBP cleavage-activating protein)-SREBP-2 complex in the endoplasmic reticulum (ER), thereby negatively regulating cholesterol biosynthesis and uptake [41]. Furthermore, oxysterols and desmosterol bind and activate LXRs, indirectly enhancing the expression of genes involved in cholesterol efflux such as ATP-binding cassette subfamily A member 1 (*ABCA1*) [42]. High cholesterol levels also prevent nuclear translocation of NRF1 and block the LXR pathway inhibition, making it a key part of the response to excess cellular cholesterol [39]. Under conditions of cholesterol deficiency, these mechanisms work together to maintain proper cholesterol levels in the cell.

## 3. Alterations of Cholesterol Metabolism in Cervical Cancer: A Clinical Approach

Cancer cells are highly proliferative and therefore heavily dependent on cholesterol to meet their increasing demand for substrates for membrane biosynthesis. Therefore, cholesterol generally support cancer by promoting oncogenic signaling, cell migration and invasion, and evasion of apoptosis [25,43,44,45].

In this manner, cancer cells improve their own cholesterol homeostasis (biosynthesis, uptake, efflux, storage), contributing to tumor progression and resistance to chemotherapeutic drugs. Consequently, reducing cholesterol or blocking its trafficking has been shown to hinder tumor growth and invasion in a variety of cancers [46,47,48,49].

Recently, the study of dysregulation of cholesterol metabolism and its effects on the incidence, progression, and clinical outcomes of patients with different types of cancer has gained interest, and some findings related to CC have been reported [19,50,51]. To date, there is evidence from studies of various cohorts that indicate an association between alterations in lipid levels, with the progression of premalignant lesions of the cervix to cancer, as well as with the clinical outcome of patients with CC. Mwangi G et al. (2024) [52] evaluated the prevalence of dyslipidemia and its association with cervical intraepithelial neoplasia (CIN) and CC in 203 women who attended at the Uganda Cancer Institute. The study showed that the prevalence of dyslipidemia in patients with CC was significantly higher (96%) than in those with CIN (73%) and those negative for intraepithelial lesions (41%). The authors also found that high levels of total cholesterol (TC) and triglycerides (TG), as well as low HDL-cholesterol (HDL-c), were associated with CIN, while elevated LDL-cholesterol (LDL-c) and TG levels were observed in CC. These findings highlight the role of dyslipidemia in the progression to CC [52].

Lin F et al. (2021) [53] analyzed 583 patients diagnosed with early-stage CC (IB1-IIA2) who underwent radical hysterectomy and pelvic lymph node dissection. They found that preoperative high levels of TC and TG, evaluated in blood serum, were associated with worse overall survival (OS), with the 5-year OS rate being 94% for low cholesterol versus 82.1% for high cholesterol levels. Therefore, the authors determined that preoperative cholesterol levels could be a potential predictor of OS in patients with early-stage CC. Similarly, Jiang Q et al. (2022) [54] analyzed the correlation between preoperative lipid levels and CC as well as its prognostic value, in a cohort that included 1713 patients with early-stage CC (I–II) undergoing radical hysterectomy and 10,397 healthy women as a control group. The results showed that CC patients had higher plasma levels of TC, TG, and LDL-c, along with lower HDL-c levels, compared to the healthy women group, regardless of age and Body Mass Index (BMI). Furthermore, those with the highest TC levels showed lower OS than those with lower TC levels, although this difference was not statistically significant [54].

In a retrospective study published by Qiu Q et al. (2024) [55], the relationship between OS rate and dyslipidemia was analyzed in a cohort with 840 patients with stages I to IV of CC. It was found that patients with elevated LDL and TC levels had significantly worse OS. Similarly, high LDL levels correlated with worse OS, disease-specific survival (DSS), and recurrence-free survival (RFS). Finally, high levels of TC, LDL, and TG, and the presence of diabetes, were independently associated with decreased OS after adjusting for age, cancer stage, and treatment type [55].

Furthermore, Cheng L et al. (2024) [56] studied a cohort of 1589 patients composed of 951 patients with clinical stage I and 638 in II–IV stages. Healthy women were also included as a control. The findings showed that, unlike healthy women, CC patients had elevated levels of TC, TG, and LDL-c along with decreased HDL-c levels. Furthermore, patients with advanced stages of CC had lower levels of HDL-c and higher levels of LDL-c than patients with early stages. Finally, the analysis highlighted those elevated levels of TC, TG, and LDL-c are significant risk factors for the development of CC [56].

Contrary to the previously described studies, Sun Y et al. (2016) [57] conducted a retrospective observational study in a cohort of 300 CC patients and 311 patients with uterine leiomyomas (benign disease). TG, TC, HDL-c, LDL-c, and lipoprotein levels were analyzed and, unlike the previously described studies, no associations of lipid profile disorders with the development of CC were found.

Overall, these studies show that in women with CC, plasma levels of TC, TG, and LDL-c are higher, and levels of HDL-c are lower than in women without cancer. Furthermore, in more advanced stages of CC, HDL-c levels are lower and LDL-c levels are higher than in less advanced stages, which is associated with reduced OS. Additionally, preoperative levels of these lipids predict patient survival. However, to strengthen these findings, it will be necessary to analyze what occurs in other populations with respect to these parameters in studies that include rigorous stratification of CC.

## 4. Alterations of Cholesterol Metabolism in Cervical Cancer: A Molecular Approach

Given the impact of altered lipid levels on the clinical outcome of patients with CC, the analysis of these elements at the molecular level deserves to be studied in depth, in order to identify possible therapeutic targets. In this regard, it has already been shown that some elements involved in cholesterol metabolism are dysregulated in CC.

For instance, the human protein encoded by the *TM7SF2* (7-transmembrane superfamily member 2) gene, responsible for reducing the delta C14 double bond of sterol intermediates in the post-squalene region of the cholesterol biosynthesis pathway, is significantly elevated in CC cell lines and tissue [58,59].

**Figure 1:**
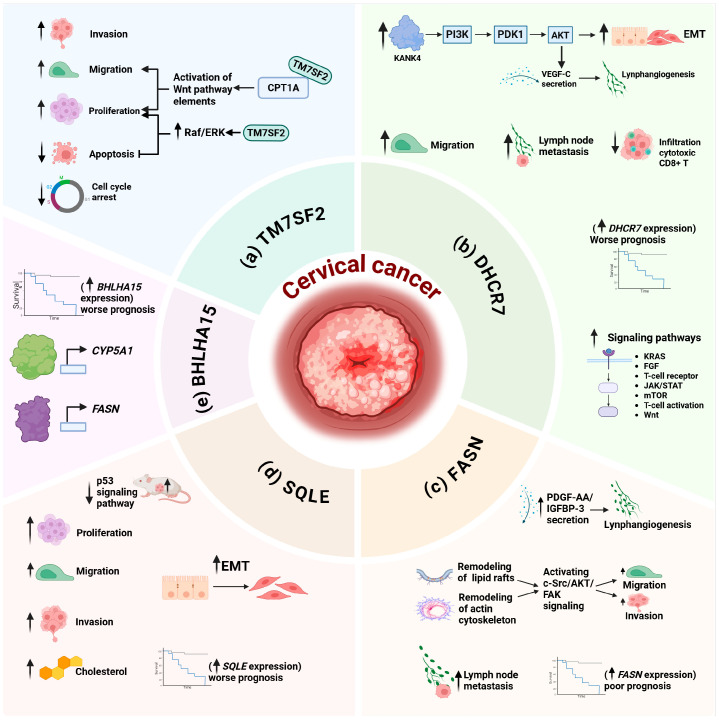
Clinical and molecular approach to cholesterol metabolism alterations in cervical cancer. (**a**) TM7SF2 promotes cell proliferation, migration, and invasion, and decreases apoptosis and cell cycle arrest. Furthermore, the binding of TM7SF2 to CPT1A activates elements of the Wnt pathway, resulting in increased cell migration and proliferation. TM7SF2 exacerbates proliferation and inhibits apoptosis via the Raf/ERK pathway. (**b**) High expression of the *DHCR7* is associated with a worse clinical prognosis in patients with cervical cancer. Furthermore, this high expression correlates with greater activation of altered signaling pathways in cancer and with lower infiltration of cytotoxic CD8+ T lymphocytes. Additionally, DHCR7 promotes epithelial–mesenchymal transition, lymphangiogenesis, and lymph node metastasis by activating the KANK4/PI3K/AKT axis and secreting VEGF-C. (**c**) High FASN levels and mRNA expression in CC patients are associated with lymph node metastasis and a poor prognosis, respectively. FASN increases cell migration and invasion by remodeling lipid rafts and the actin cytoskeleton, and subsequently by activating the c-Src/AKT/FAK signaling pathway. FASN accelerate lymphangiogenesis by increasing PDGF-AA/IGFBP-3 secretion. (**d**) High expression of the SQLE is associated with reduced survival in patients with cervical cancer. Furthermore, SQLE promotes epithelial–mesenchymal transition, proliferation, migration, invasion, and evasion of the immune system. Additionally, elevated SQLE expression increases cholesterol levels and decreases p53 expression, which exacerbates cell proliferation and tumor growth. (**e**) High expression of *BHLHA15* correlates with a poor clinical prognosis in patients with cervical cancer. Furthermore, BHLHA15 enhances the transcription of *CYP51A1* and *FASN*, which affects the reprogramming of cholesterol metabolism and, consequently, promotes the progression of cervical cancer. Created in BioRender. Martínez Ramírez, I. (2026) https://BioRender.com/ymp8ajv (accessed on 2 January 2026).

It was also reported that in CC cell lines, increased levels of TM7SF2 had a positive effect on proliferation, migration, and invasion, while negatively impacting on cell apoptosis and G0/G1 phase arrest (Figure 1a). Functional assays using CC cell lines demonstrated that the mechanism by which TM7SF2 induced cervical oncogenesis involves the c-Raf/ERK signaling pathway, which promotes cell proliferation and inhibits apoptosis. Finally, through in vivo model assays revealed that TM7SF2 contributes to the progression of CC by promoting tumor growth and inhibiting apoptosis (Figure 1a) [59].

Additionally, it was shown in CC cell lines that cell migration and proliferation promoted by TM7SF2 can also be regulated by means of its direct binding to the enzyme Carnitine palmitoyl transferase 1 A (CPT1A), which controls the entry of fatty acids into the mitochondria for their oxidation. In turn, CPT1A regulates the Wnt/β-Catenin pathway; therefore, another mechanism by which TM7SF2 regulates cell proliferation and migration in CC cell lines is through the induction of lipid reprogramming by the CPT1A/Wnt/β-catenin axis (Figure 1a) [60].

Another key enzyme in cholesterol biosynthesis overexpressed in CC is 7-dehydrocholesterol reductase (DHCR7), which regulates the rate of cholesterol synthesis through the removal of the C (7-8) double bond in the B ring of sterols, which reduces 7-dehydrocholesterol to cholesterol [61].

Zou J et al. (2022) [62] analyzed a dataset of CC patients from the Cancer Genome Atlas (TCGA) and found that those patients with high *DHCR7* gene expression had a worse clinical outcome in terms of OS, Progression-Free Interval (PFI), and DFS, compared to those patients who had low *DHCR7* levels. Consequently, overexpression of *DHCR7* could be considered a biomarker of poor prognosis for CC patients. The results of this analysis showed that increased *DHCR7* gene expression correlated with an enrichment of signaling pathways such as KRAS, FGF, T-cell receptor, JAK/STAT, mTORC1, T-cell activation, and the Wnt-activated receptor. Moreover, cellular processes related to G2/M checkpoint, macrophage migration, and regulation of cholesterol biosynthesis were exacerbated. In addition, *DHCR7* expression inversely correlated with tumor-infiltrating lymphocytes. Finally, it was observed that DHCR7 protein levels were significantly higher in CC cell lines than in non-tumor epithelial cell lines and that downregulation of DHCR7 protein negatively affected cell proliferation (Figure 1b). Therefore, DHCR7 could participate in CC development and progression [62].

In vitro experiments in CC cell lines showed that overexpression of DHCR7 promotes epithelial-to-mesenchymal transition and lymphangiogenesis; meanwhile in vivo assays, DHCR7 overexpression is associated with metastasis to lymph nodes [63]. Furthermore, it was reported that the mechanism by which DHCR7 induces these processes in CC is through the activation of the KANK4/PI3K/AKT axis and secretion of VEGF-C, in a manner dependent on the reprogramming of cholesterol metabolism (Figure 1b) [63]. It was found that DHCR7 reprograms cholesterol metabolism, thereby increasing KANK4 levels and alternatively, promoting VEGF-C secretion, which is associated with the induction of lymphangiogenesis. Consequently, KANK4 activates the PI3K/AKT signaling pathway, which promotes epithelial–mesenchymal transition, cell migration, and invasion. In this work, it was also demonstrated that CC patients with high *DHCR7* expression had significantly lower DFS and OS than those with low levels (Figure 1b) [63].

It is worth noting that the fatty acid synthase enzyme (FASN), responsible for catalyzing the synthesis of long-chain fatty acids, also participates in cholesterol synthesis [64]. It was shown that acetoacetyl-CoA produced by FASN activity is a key metabolite in cholesterol production. In this sense, Du Q et al. (2022) [65] found that in patients with CC, there is a positive correlation between elevated FASN levels and lymph node metastasis, and high expression of *FASN* was correlated with a poor prognosis (Figure 1c). The authors demonstrated that CC cell lines as well as CC tissue samples presented elevated levels of FASN. Furthermore, in vitro experiments showed that FASN increases cell migration and invasion, primarily by reprogramming cholesterol metabolism, causing remodeling of lipid rafts and the actin cytoskeleton, and subsequently by activating the c-Src/AKT/FAK signaling pathway. It was found that FASN can accelerate lymphangiogenesis by upregulating the secretion of PDGF-AA/IGFBP-3 (Figure 1c). Finally, in an in vivo model, it was revealed that elevated levels of FASN contribute to the development of metastases in CC, as evidenced by an increase in the percentage of lymph node metastases, a higher density of lymphatic structures, and increased lymphatic volume [65].

Regarding cholesterol synthesis, after 3-hydroxy-3-methylglutaryl-CoA reductase (HMGCR), squalene epoxidase (SQLE) is the second rate-limiting enzyme. SQLE, also known as squalene monooxygenase, is responsible for the first oxidation reaction that converts squalene to 2,3-epoxysqualene, and when lanosterol synthase enzyme levels are low, SQLE can convert 2,3-epoxysqualene into dioxidesqualene [66]. The final product of this shunt pathway, 24(S),25-epoxycholesterol, functions as a ligand for the hepatic X receptor (LXR). This interaction upregulates the ATP-binding cassette transporter protein A1 (ABCA1), thus promoting cholesterol production [66]. Interestingly, using bioinformatics analysis, Guo M et al. (2023) [67], determined that CC tissues exhibited high levels of SQLE, compared to adjacent tissue, which correlated with patient’s shorter OS. Furthermore, they confirmed that in both CC-derived cell lines and CC tissues, *SQLE* expression and protein levels were significantly higher than in a cancer-free cervical cell line and adjacent tissue, respectively. Molecular assays determined that SQLE induced cell proliferation, migration, and invasion in CC-derived cell lines. Finally, in vivo assays showed that SQLE overexpression increased tumor growth, mediated by a decrease in the tumor suppressor protein p53, corroborating the findings of the bioinformatics analyses.

Zhao Y et al. (2024) [68] reported that SQLE overexpression may be involved in the growth and aggressiveness of CC tumors. *SQLE* expression levels were analyzed in different types of cancer from the Timer 2.0 pan-cancer database, it was found that CC patients’ tissues had considerably higher *SQLE* gene expression than healthy patients’ tissues. When evaluating the diagnostic capacity of *SQLE* expression levels, the authors determined that differential SQLE levels could serve as a powerful diagnostic tool for distinguishing between normal patients and those with CC, as well as for identifying tumor stages. In this study high levels of *SQLE* gene expression in stages T3/T4 were strongly associated with lower survival rates. Using in vitro and in vivo functional assays, it was demonstrated that SQLE can promote epithelial–mesenchymal transition (a key process in tumor metastasis), proliferation, migration, invasion, and evasion of the immune system, as well as affecting tumor size and aggressiveness, thus participating in the development of CC. It was also demonstrated that *SQLE* gene expression levels are directly related to cholesterol levels, which in turn correlated with an exacerbated cell proliferation (Figure 1d) [68].

Finally, it has been reported that dysregulation of transcription factors in cervical cancer may affect cholesterol homeostasis, promoting tumor progression. Wang W et al. (2021) [69], reported that the transcription factor basic helix-loop-helix family member a15 (BHLHA15), also called MIST1, an important regulator in cellular processes such as organogenesis, regulates tumor progression by inducing epithelial–mesenchymal transition in some cancers, including CC. Comparative analysis of *BHLHA15* gene expression levels in normal tissue and CC primary tumors showed that *BHLHA15* expression was higher in CC primary tumors. Furthermore, the authors determined that elevated expression of *BHLHA15* correlated with lower OS and DFS (Figure 1e) [69]. Recently, Ye H et al. (2025) [70], through bioinformatics analysis and subsequent validation with in vitro and in vivo assays as well as in CC tissues, determined that the expression levels of *BHLHA15* and its transcriptional targets *CYP51A1* and *FASN* are elevated in CC cell lines and tissue, with respect to normal cervical cells and adjacent normal cervical tissue, respectively (Figure 1e). It was found that high expression of *BHLHA15* in CC was strongly associated with stages III–IV, mortality, and poor prognosis. Finally, the authors reported that BHLHA15, by directly activating the transcription of *CYP51A1* and *FASN*, acts as a critical transcriptional factor in regulating cholesterol metabolism reprogramming, favoring CC progression (Figure 1e) [70].

## 5. Regulation of Cholesterol Metabolism by HPV Oncoproteins

The role of E6 and E7 oncoproteins in the dysregulation of cellular energy pathways has been extensively studied [71]. However, there is limited information on their direct participation in cholesterol metabolism and their potential contribution to carcinogenesis, maintenance of the tumor phenotype, or progression of HPV-related tumors. Comprehensive omics analysis suggests that E6 and E7 may contribute to increased cholesterol metabolism. To better understand the effects of the viral oncoproteins, the list of genes deregulated by HPV-16 E6 and E7 oncoproteins from the different studies described below was compared with the set of genes included in the term cholesterol metabolic process from the Gene Ontology annotations [72]. Recently, our group demonstrated that in cervical cancer C-33 A cells, HPV-16 E6 oncoprotein altered the expression of 22 cholesterol metabolism-related genes, 15 of which were overexpressed and 7 underexpressed; whereas E7 oncoprotein deregulated the expression of 23 genes, 15 of which were overexpressed and 8 downexpressed. Interestingly, 8 genes were upregulated by both oncoproteins (*EBP*, *FDFT1*, *GNB3*, *LDLR*, *LIMA1*, *LRP5*, *SCARB1*, and *TTC39B*), while 5 genes were downregulated by both oncoproteins (*CUBN*, *FGF1*, *PMP22*, *SC5D*, and *TNFSF4*) (Table 1) [73].

The expression of these genes and the protein levels of some of them have been previously linked to cancer. For example, farnesyl diphosphate farnesyltransferase 1 (FDFT1) also known as squalene synthase, is an enzyme that synthesizes squalene by condensing two farnesyl pyrophosphate molecules. FDFT1 has been reported to induce metabolic reprogramming, cell proliferation, and invasion. Overexpression of *FDFT1* is required to produce cholesterol precursors, which are necessary for tumor progression, lipid rafts formation, signal transduction, and cancer cell proliferation, invasion, and migration [74]. Another gene that is downregulated in cells with E6 and E7 expression is sterol-C5-desaturase (*SC5D*), an enzyme that catalyzes the conversion of lathosterol into 7-dehydrocholesterol [75]. It has been postulated that a reduction in SC5D activity in cancer could promote the prenylation of Ras, Rac or RhoC, impacting cancer progression [76].

When osteosarcoma-derived cells, U2OS, were stably transfected with HPV-16 E6, few genes related to cholesterol metabolism were altered, among which *HMGCS1*, *LBR*, and *PRKAA1* were upregulated, whereas only one gene, *FDXR*, was underexpressed [77].

On the other hand, in immortalized keratinocytes HaCaT, with stable expression of HPV16 E6/E7 oncogenes, 45 genes related to cholesterol metabolism was deregulated, with 27 genes being overexpressed while 18 genes were downregulated [78] (Table 1).

In a proteomic analysis study performed in primary human neonatal keratinocytes (HEKn cells) with stable expression of HPV-16 E6/E7, it was found that the protein products of the *LBR* and *VDLR* genes increased, while those of *APLP2*, *APOE*, *CLN8*, *LDLR*, *LIPE*, *NPC2*, *NSDHL*, *SC5D*, and *SCAP* decreased in relation to cells transfected with the empty vector [79]. Furthermore, in immortalized female human oral keratinocytes (NOKs) stably expressing HPV-16 E6/E7 oncogenes, transcriptomic and proteomic analysis showed that both the expression and protein levels of *PMVK* were increased, while *SAA1*, *NPC2*, and *SEC14L2* decreased, compared with the control cell group [80].

Notably, the deregulated genes detected in various studies merit further study to analyze the effect of viral oncoproteins on cholesterol metabolism, both in cancer and in an infectious context. Below, some of the genes mentioned in different studies are highlighted.

As previously mentioned, in U2OS and HEKn cells, expressing E6 and E6/E7, respectively, LBR gene expression was upregulated. Interestingly, the *LBR* gene encodes the lamin B receptor, a polytopic membrane protein located in the inner nuclear membrane, associated with the nuclear lamina. The C-terminal domain has been shown to anchor lamin B receptor to the inner nuclear membrane. Furthermore, it exhibits sequence homology with sterol C14 reductases, so lamin B receptor participates in cholesterol synthesis, catalyzing the reduction in the C14-unsaturated bond of lanosterol [81]. The involvement of lamin B receptor in promoting cell proliferation and immortalization in p53- and RB-deficient cells has been previously identified [82].

The *NPC2* (Niemann-Pick Disease Type C2) gene encodes a protein with a lipid-recognition domain, which is involved in cholesterol transport across the late endosomal/lysosomal system. NPC2 is involved in the export of cholesterol derived from lysosomal hydrolysis of low-density lipoproteins. NPC2 has been shown to accelerate sterol exchange between liposomes, especially under conditions that mimic the intra endosomal environment [83]. The *NPC2* gene has been previously described as part of a group of lysosomal-associated genes in lung adenocarcinoma. These genes were weakly expressed in malignant epithelial cells, and patients with low expression showed a higher percentage of M2 macrophage infiltration. Furthermore, restoring the expression of the lysosomal-associated genes signature, including NPC2, diminished malignant cell proliferation and invasion and reduced M2 macrophage polarization and cellular secretion of pro-tumor cytokines [84]. In contrast, the *NPC2* gene was found to be significantly upregulated in gliomas; patients with high *NPC2* expression had higher levels of immune cell infiltration and were associated with poor clinical outcome [85]. Interestingly, oncogenes E6 and E7 reduced *NPC2* expression in HEKn and NOKs cells but promoted its overexpression in HaCaT cells.

Two genes encoding receptors involved in cholesterol entry are *LDLR* and *SCARB1*, which recognize LDL and HDLs, respectively. Both E6 and E7 have been shown to overexpress *LDLR* and *SCARB1* in C-33A cells. Previous studies using murine models have demonstrated that mammary tumors with high *LDLR* expression increased in size in mice with elevated serum LDL-c levels [86]. Furthermore, increased *SCARB1* expression has been observed in primary prostate cancer. In a castration-resistant prostate cancer (CRPC) cell model, SCARB1 inhibition suppressed cholesterol uptake, cell growth, and viability, and induced endoplasmic reticulum stress and autophagy [87]. Therefore, HPV oncoproteins E6 and E7 may promote the entry of cholesterol into cancer cells, a hypothesis that needs to be investigated.

The *CUBN* gene encodes cubilin, a high-affinity receptor responsible for the endocytosis of HDL-C and lipid-poor apoA-I, and functions as a receptor for intrinsic factor-vitamin B12 complexes [88,89]. Its overexpression has been detected in samples from patients with colorectal cancer, with higher levels of *CUBN* in advanced stages. In contrast, *CUBN* levels are low in patients who have undergone radiation therapy. Furthermore, high *CUBN* expression has been associated with a worse prognosis (recurrence and survival) in patients with colorectal cancer [90]. Transcriptomic data from HaCaT cells harboring E6/E7 expression and in C-33 A cells with E6 and E7 expression showed a decrease in *CUBN* expression, which could affect vitamin B12 absorption and HDL entry.

The *PRKAA1* gene encodes the α1 catalytic subunit of AMP-activated protein kinase, a catalytic subunit of 5′-AMP-activated protein kinase (AMPK), that regulates cellular energy by modulating glucose and lipid metabolic pathways [91]. In gastric cancer cells, *PRKAA1* silencing significantly inhibits proliferation and promotes cell cycle arrest and apoptosis, in addition to reducing tumor growth in mouse models. Furthermore, this study revealed that PRKAA1 protumoral activity is mediated by the JNK1 and/or AKT signaling pathways [92]. In addition, it was observed that patients with gastric cancer with distant metastasis and advanced stages showed increased *PRKAA1* expression [93]. Specifically, the E6 oncoprotein induces *PRKAA1* overexpression in cancer cells such as C-33A and U-2OS cells, whereas coexpression of E6 and E7 decreases *PRKAA1* expression.

Interestingly, the *SREBF2* gene is overexpressed in C-33 A cells expressing E7 [73]. Its protein product, SREBP-2, plays an important role in regulating lipid homeostasis [94]. SREBP-2 target genes include those encoding rate-limiting enzymes in cholesterol biosynthesis pathways, as well as proteins that mediate cholesterol entry, such as LDLR and SRB1 [94]. Activation of the PI3K/AKT/mTORC1 signaling pathway is known to induce proteolytic cleavage of SREBP and its nuclear translocation [95]. It is possible that the E6 and E7 oncoproteins promote its nuclear translocation and transcriptional activity, given that both have been previously shown to induce mTORC1 activation [96,97].

**Table 1:** Deregulation of genes and proteins related to cholesterol metabolism by HPV E6 and E7 oncogenes. Black arrows pointing downwards indicate that the genes/proteins are downregulated, while arrows pointing upwards indicate that they are upregulated.

Oncoprotein	Model	Biomolecule	Level	Gene or Protein	References
E6	C-33 A cells	mRNA	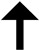	*APP*, *EBP*, *FDFT1*, *FDX1*, *GNB3*, *LDLR*, *LIMA1*, *LRP5*, *MAPK1*, *PRKAA1*, *SCARB1*, *SOAT1*, *SOD1*, *STARD4*, *TTC39B*	[73]
			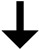	*CUBN*, *FGF1*, *SC5D*, *TM7SF2*, *TNFSF4*, *LRP1*, *PMP22*	
	U-2 OS cells	mRNA	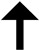	*HMGCS1*, *LBR*, *PRKAA1*	[77]
			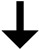	*FDXR*	
E7	C-33 A cells	mRNA	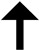	*DHCR24*, *LIMA1*, *LDLR*, *FDFT1*, *LRP5*, *DHCR7*, *SREBF2*, *SCARB1*, *LSS*, *EBP*, *TTC39B*, *DGAT2*, *ABCA2*, *INSIG1*, *GNB3*	[73]
			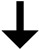	*CUBN*, *CYP39A1*, *TNFSF4*, *FGF1*, *LEPR*, *PCTP*, *SC5D*, *PMP22*	
E6/E7	HaCaT cells	mRNA	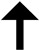	*ABCA5*, *APLP2*, *CEBPA*, *CLN8*, *CYP7B1*, *DHCR24*, *EPHX2*, *FDFT1*, *FDXR*, *HMGCS1*, *HSD17B7*, *LBR*, *LDLRAP1*, *LMF1*, *MVD*, *MVK*, *NPC2*, *PCSK9*, *PCTP*, *SAA1*, *SEC14L2*, *SOAT2*, *SQLE*, *SULT2B1*, *THRB*, *TM7SF2*, *TTC39B*	[78]
			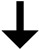	*ABCA1*, *ABCA2*, *ABCG1*, *ACADL*, *CUBN*, *CYB5R3*, *GNB3*, *HSD3B7*, *LEPR*, *LRP5*, *NPY1R*, *PMP22*, *PMVK*, *PRKAA2*, *SCAP*, *SCARB1*, *SMPD1*, *VLDLR*	
	HEKn cells	Protein	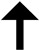	LBR, VDLR	[79]
			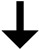	APLP2, APOE, CLN8, LDLR, LIPE, NPC2, NSDHL, SC5D, SCAP	
	NOKs Cells	mRNAs and their protein products	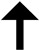	*PMVK*	[80]
			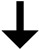	*NPC2*, *SEC14L2*, *SAA1*	

Accumulating evidence suggests that HPV E6 and E7 oncoproteins can regulate cholesterol metabolism. Omics studies are a valuable source of information that allows, as a first step, the establishment of a causal association between the possible involvement of viral oncoproteins and altered cholesterol metabolism. While current omics studies are limited, further in-depth research is needed on the direct role of the E6 and E7 oncoproteins in the dysregulation of cholesterol metabolism and their biological impact on tumor transformation or progression. It is worth noting that the aforementioned evidence comes from high-throughput sequencing and proteome analysis, where the exogenous expression of E6 and/or E7 allows for exploring changes in the transcriptome and proteome, with their potential impact on exacerbating cellular processes. However, validations in in vitro and in vivo assays are required to confirm the direct involvement of viral oncoproteins in cholesterol metabolism.

Table 1 summarizes genes and/or proteins related to cholesterol metabolism altered in the presence of HPV E6 and E7 oncogenes.

## 6. Cholesterol Metabolism as a Therapeutic Opportunity in Cervical Cancer

Reprogramming of cholesterol metabolism is crucial for cancer development and its maintenance. Preclinical and clinical studies have demonstrated that modulating cholesterol metabolism can slow tumor growth, alter the immune environment, and enhance antitumor immune responses. As a result, various approaches have been developed to target cholesterol metabolism to combat cancer. These strategies include repurposed drugs and specific inhibitors targeting different stages of cholesterol metabolism (biosynthesis, uptake, efflux, and storage) [98].

Developing new anticancer drugs is expensive and involves a long research process [99]. As a result, efforts have been made to repurpose pharmacological treatments approved for other diseases for cancer therapy. Such is the case of statins, which are competitive inhibitors of HMGCR and have been used for cholesterol lowering. These drugs bind to the active site of HMGCR, inhibiting the conversion of HMG-CoA to mevalonic acid, which is an early step in cholesterol synthesis [100,101]. Lipid-lowering agents are widely used to prevent cardiovascular disease [102,103]. Since the early 1990s, studies have shown that statins not only lower cholesterol levels but also inhibit tumor growth [104,105], and their use is associated with a reduced risk of gynecological cancers such as endometrial and ovarian cancer [106] and with lower mortality in these types of cancer. In CC, simvastatin has been shown to enhance in vitro and in vivo efficacy of chemotherapy [107].

Statins can be categorized into two groups based on their chemical structure: hydrophilic (pravastatin and rosuvastatin) and lipophilic (cerivastatin, simvastatin, lovastatin, fluvastatin, and atorvastatin). Biochemical differences between these two types may affect their uptake mechanisms [108]. In preclinical models, combining simvastatin with paclitaxel (a chemotherapeutic agent against CC) significantly improves paclitaxel effectiveness [107]. Phase II clinical trials (identification numbers NCT03324425 and NCT00313859) indicate that some patients with different types of cancer might benefit from combining simvastatin with other chemotherapeutic drugs [109,110].

Research has shown that prescribing statins for more than one year before diagnosis reduces the risk of endometrial and ovarian cancer. In addition, women who used statins only after a cancer diagnosis had significantly better survival rates [111]. Similarly, in another cohort study by Habis M et al. (2014) [112] it was found that patients with non-serous papillary epithelial ovarian cancer and hyperlipidemia who were treated with statins experienced increased DSS and PFS (Progression-Free Survival).

Few studies have examined the use of statins in the risk of CC, and their results are controversial [113,114,115]. Liu Y et al. (2014) [113] performed a meta-analysis on the impact of statins on CC risk, which was inconclusive due to the limited number of studies and small case numbers in each one. Recently, Kim D et al. (2022) [116] compared women who used oral statins for at least 6 months with a control group. Their findings indicated that statin use was significantly associated with a lower risk of developing breast cancer and CC incidence. Conversely, Jiao X et al. (2024) [115] reported that statin use significantly increases the risk of CC in a UK cohort study.

Regarding the role of statins in the clinical outcomes of patients with CC, Song M et al. (2017) [114] found that patients diagnosed with locally advanced cervical cancer (LACC) who consistently used lipophilic statins for a prolonged period before their CC diagnosis had significantly longer PFS and OS compared to the control group. Additionally, they showed that the adverse effect of high BMI on clinical outcomes in patients with CC can be reduced by treatment with lipophilic statins. The same study also indicated that hydrophilic statins had a non-significant protective effect, possibly because lipophilic statins can penetrate extrahepatic cells and, thus, reach higher concentrations in all tissues; in contrast, hydrophilic statins tend to be more hepatoselective [108,117].

FASN-derived acetoacetyl-CoA is vital for cholesterol synthesis, which favors the formation of lipid rafts that promote the pro-inflammatory response by regulating TLR signaling (Toll-like receptor) [64]. High FASN levels promote cell proliferation, invasion, tumor progression, and drug resistance in female cancers, including breast cancer [118,119,120,121] ovarian cancer [122,123] and CC [124,125,126] FASN expression was progressively higher in cervical low-grade squamous intraepithelial lesions (LSIL) and in high-grade squamous intraepithelial lesions (HSIL), and CC samples, indicating a possible role of this enzyme in cervical carcinogenesis [124]. It has been reported that FASN is overexpressed in CC, leading to reprogrammed cholesterol metabolism and increased lymph node metastasis, both of which are associated with a poor prognosis in CC patients [65,125].

Inhibitors of FASN activity have been evaluated in cancer, including cerulenin, C75, orlistat (ORL), and TVB-264, but cerulenin and C75 have serious side effects in animal models [126,127,128]. TVB-2640 (Denifanstat) is a recently added inhibitor in clinical trials with promising results [129,130]. TVB-2640 has demonstrated potent effects as monotherapy and in combination with paclitaxel and bevacizumab across various solid tumors, including breast cancer, non-small cell lung cancer, and CC (identification numbers NCT02223247, NCT03032484 and NCT03808558) [110,129]. Additionally, Wang X et al. (2025) [131] found that FASN overexpression correlated with cisplatin resistance in CC and showed that TVB-2640 significantly improved the effectiveness of cisplatin therapy, markedly suppressing the growth of xenograft tumors derived from cisplatin-resistant CC cells.

Orlistat (ORL) is an irreversible FASN inhibitor because it binds the thioesterase domain, which blocks palmitate synthesis [132]. Moreover, it inhibits the pancreatic lipase in the gastrointestinal tract, for which it has been approved for the treatment of obesity [133]. Furthermore, ORL has demonstrated antitumor effects and enhances therapy in various tumor types [134,135,136,137,138]. In CC-derived cell lines, inhibition of FASN with ORL decreased cell viability, reduced colony formation, and induced apoptosis and cell cycle arrest [124]. ORL has drawbacks, including low water solubility and poor oral bioavailability, which limit its formulation and clinical use; pharmacological efforts have been made to improve its solubility and enhance its therapeutic potential for cancer [139,140].

The debate surrounding the use of inhibitors of key cholesterol-metabolizing proteins for CC remains highly controversial, largely due to methodological limitations. Specifically, in the case of statins, some studies, including those discussed in this section, suggest that statins may reduce the risk of developing CC; however, this effect is not yet conclusive. The primary source of this debate is the inconsistent findings across studies. These discrepancies may stem from variations in study populations; differences in statin dosage and duration of use and differences in statin physicochemical properties (lipophilic vs. hydrophilic). Evidence suggests that statins with distinct physicochemical properties may exert anticancer effects through different mechanisms, independent of their lipid-lowering pharmacological effects. For example, lipophilic statins have been shown to have greater pro-apoptotic and cytotoxic activity than hydrophilic statins, such as pravastatin [141].

Moreover, the emerging evidence that suggests that statins may play a role in the treatment of various cancers by synergizing with immunotherapy and certain chemotherapeutic agents [142,143,144], should be approached with caution, since adding atorvastatin to gemcitabine treatment has been shown to reduce the efficacy of chemotherapy in patients with pancreatic cancer [145]. The evidence supporting its use in the treatment of CC is encouraging but limited; therefore, future research should focus on establishing the dose–response relationship and identifying the most effective dosing regimens for the treatment of patients with CC, designing personalized treatment and exploring combining statins with other anticancer therapies as immunotherapy. Addressing these current controversies and optimizing statin use will be crucial for their successful application in cervical cancer treatment.

## 7. Concluding Remarks

Energy reprogramming is a necessary condition that enables cancer cells to survive in hostile environments, such as hypoxia, nutrient deprivation, or changes in extracellular acid-base conditions. In different tumor types, changes in the energy circuits associated with cell survival and progression have been studied. In particular, in CC, a tumor frequently associated with persistent HPV infection, dysregulation of glucose, protein, and lipid metabolism has been described [146,147]. Several approaches have been used to associate serum cholesterol levels to the clinical course of CC patients, demonstrating its role in disease development and progression. Furthermore, some elements of the catabolic and anabolic cholesterol pathways that are altered in cancer have been identified, and their association with patient prognosis has been established. While there is limited evidence regarding the involvement of HPV oncoproteins E6 and E7 in the dysregulation of cholesterol metabolism, the emergence of new omics technologies has focused attention on certain genes or proteins whose regulation is altered by the viral oncogenes. These dysregulations could exacerbate cholesterol uptake and metabolism and, in turn, alter other signaling pathways that, together, exacerbate cancer-related processes such as proliferation, migration, invasion and metastasis, among others. High expression of HPV oncoproteins has been previously described as being associated with a poor clinical prognosis of patients with CC [148,149]. Therefore, further studies are needed to elucidate the specific role of viral oncoproteins in regulating cholesterol metabolism, in order to identify biomarkers with prognostic value and molecules that could serve as pharmacological targets. In this review, we highlight the strategy of controlling lipid metabolism for combating cervical tumors and, consequently, improving the quality of life of patients. Cholesterol-derived metabolites promote cancer progression and suppress immune responses. Preclinical and clinical studies have demonstrated that strategies targeting cholesterol metabolism suppress tumor growth, alter the immune microenvironment, and activate antitumor immunity [46]. In this regard, the pharmacological strategies studied to date have shown promising results, but with limited effect in preclinical studies. Therefore, it is necessary to continue investigating the molecular mechanisms underlying cancer aggressiveness, including lipid metabolism in CC, in order to develop effective therapeutic strategies for this disease.

## Data Availability

No new data were created or analyzed in this study. Data sharing is not applicable to this article.
